# The dual face of NAMPT: Intracellular/extracellular protein and diagnostic/therapeutic target in cancer

**DOI:** 10.1016/j.ebiom.2020.103109

**Published:** 2020-11-05

**Authors:** Valentina Audrito

**Affiliations:** Laboratory of Tumor Immunogenetics, Department of Medical Sciences, University of Turin, Turin, Italy

Since the discovery of nicotinamide phosphoribosyltransferase (NAMPT) as a pre-B cell-enhancing factor in 1994 by Samal et al. [1], many papers have described the pleiotropic function of this enzyme. A vital role of NAMPT in mammalian cells was attributed to its activity as a rate-limiting enzyme in the biosynthesis of nicotinamide adenine dinucleotide (NAD) from nicotinamide, and was supported by its widespread tissue distribution and by the embryonic lethality of total *NAMPT* knock-out mice [2].

It has been shown that tumours require increased levels of energetic molecules. Metabolic rewiring is now considered one of the hallmarks of tumours, sustaining the production of cellular energy [ie, adenosine triphosphate (ATP)], and their growth and invasive capacities [3]. The redox co-factor NAD, is fundamental in several metabolic reactions, and as a substrate for different NAD-consuming enzymes [eg, poly-ADP-ribose polymerases (PARPs) and sirtuins], involved in many cellular processes, including DNA repair and epigenetic regulation of gene expression [4]. A common strategy that several tumour types adopt to sustain NAD production is to over-express NAMPT, regulated at the transcriptional level [5]. This finding has led to the design of numerous NAMPT inhibitors (NAMPTi) in the context of cancer therapy. Despite significant results *in vitro* and *in vivo*, phase I clinical trials with the first NAMPTi (ie, FK866 and GMX1778) in haematological or solid malignancies, showed no objective tumour remission, and toxicity. Besides the pharmacological properties of these drugs and their potential toxicity, one of the main aspects leading to the partial failure of NAMPTi treatment was due to the concomitant expression of others NAD-biosynthetic enzymes that can overcome NAMPT inhibition. It is now clear in the field that it is necessary to select tumours uniquely addicted to NAMPT activity in the generation of NAD. Novel NAMPTi are currently in pre-clinical and phase I-II clinical trials, highlighting an intense pharmacological effort to target this enzyme [6].

The initial rational to develop NAMPTi was based solely on the role of NAMPT as an intracellular NAD-biosynthetic enzyme. However, NAMPT has emerged as a mediator of inflammation with important, context-dependent, extracellular functions [7]. Increased extracellular (e)NAMPT levels are reported in conditions of acute or chronic inflammation, including tumours, correlating with worse prognosis and increased tumour aggressiveness as reviewed in [7]. Moreover, eNAMPT acts as a cytokine that modulates the immune response, and as an adipokine (also known as visfatin) that plays a critical role in metabolic diseases [5]. In 2015, Garcia and colleagues identified Toll-like receptor 4 (TLR4) as an eNAMPT receptor, therefore adding the enzyme to a number of damage-associated molecular patterns (DAMPs) molecules: “danger” signals activating this receptor [7,8]. Much unknown and incomplete information remains to be addressed regarding the nature of eNAMPT's post-translational modifications, its mechanism of secretion, and its enzymatic activity in the extracellular space. However, a proposed strategy of blocking eNAMPT is emerging. This is the focus of the paper by Sun et al. published in this issue of *EBioMedicine* [9]. The tumour model that the authors used to verify the role of eNAMPT as a biomarker and therapeutic target was invasive prostate cancer (PCa). Over-expression of intracellular NAMPT protein was previously demonstrated in 2011 by Wang et al. They demonstrated that inhibition of NAMPT significantly suppresses cell growth in culture, soft agar colony formation, cell invasion, and growth of xenografted prostate cancer cells in mice [10]. However, the role of NAMPT in PCa is largely unexplored. Sun et al. added novel insight about i) the regulation of NAMPT expression in PCa via hypoxia-inducible factor 1-alpha/beta (HIF-1α/β), ii) the presence of higher eNAMPT protein in plasma from PCa patients compared to healthy donors, iii) the activity of eNAMPT in supporting the invasive features of PCa, and iv) the tumour blocking activity of anti-eNAMPT neutralizing antibody in a pre-clinical *in vivo* model of PCa invasion. The regulation of *NAMPT* transcriptional expression and the activity of eNAMPT in promoting a tumour invasive phenotype are known in a variety of tumour types. However, two novel and significant findings emerged from this paper.

The first one is that the elevated levels of eNAMPT in PCa have a predictive value for PCa diagnosis and correlate with aggressive PCa, suggesting that this protein could be consider a novel biomarker in PCa. This finding opens up the possibility of a clinically-utilized eNAMPT biomarker screen to assess PCa risk. To-date, prostate-specific antigen (PSA) levels are considered the only PCa biomarker to discriminate between prostate benign hyperplasia or inflammation and PCa. However, PSA assays, as discussed by the authors, suffer from high false-positive results, high variability, and fails to correlate with PCa aggressiveness. On the contrary, at least in the cohort analyzed in the paper, high (>30 ng/ml) eNAMPT levels are associated with aggressive and invasive PCa. Of course, future studies to confirm these results are needed. However, this paper proposes, for the first time, eNAMPT as a biomarker in PCa patients, to be used alone or in combination with PSA.

The second important and translational message is the therapeutic potential of eNAMPT neutralization, using a specific blocking antibody, to prevent the invasiveness and metastasis of PCa. The authors studied the capacity of PCa cells to invade the inferior diaphragmatic muscle *in vivo*, demonstrating that mice treated with polyclonal neutralizing anti-eNAMPT antibody had fewer and smaller colonies on the inferior diaphragm, confirming an inhibition of invasive capacity. The general idea of targeting eNAMPT in tumours is increasing, to counteract the extracellular functions of this protein, mainly linked to the activation of TLR4 and modulation of immune responses. It would be very interesting to address whether the block of eNAMPT acts on the PCa tumour as well as on the PCa tumour microenvironment. This approach might not be restricted to PCa, and perhaps a combination of pharmacological NAMPTi and eNAMPT blocking antibody to affect the dual roles of this enzyme/cytokine, will be a successful approach to halt tumour progression and instigate a less supportive tumour microenvironment.

## Contributors

V.A. contributed to the literature search, data analysis, data interpretation, and writing.

## Declaration of Competing Interests

Dr. Audrito has no conflicts of interest to disclose.

## References

[1] Samal B, Sun Y, Stearns G, Xie C, Suggs S, McNiece I (1994). Cloning and characterization of the cDNA encoding a novel human pre-B-cell colony-enhancing factor. Mol Cell Biol.

[2] Revollo JR, Korner A, Mills KF, Satoh A, Wang T, Garten A (2007). Nampt/PBEF/Visfatin regulates insulin secretion in beta cells as a systemic NAD biosynthetic enzyme. Cell Metab.

[3] Pavlova NN, Thompson CB. (2016). The emerging hallmarks of cancer metabolism. Cell Metab.

[4] Chiarugi A, Dolle C, Felici R, Ziegler M (2012). The NAD metabolome–a key determinant of cancer cell biology. Nat Rev Cancer.

[5] Garten A, Schuster S, Penke M, Gorski T, de Giorgis T, Kiess W (2015). Physiological and pathophysiological roles of NAMPT and NAD metabolism. Nat Rev Endocrinol.

[6] Galli U, Colombo G, Travelli C, Tron GC, Genazzani AA, Grolla AA (2020). Recent advances in NAMPT inhibitors: a novel immunotherapic strategy. Front Pharmacol.

[7] Audrito V, Messana VG, Deaglio S (2020). NAMPT and NAPRT: two metabolic enzymes with key roles in inflammation. Front Oncol.

[8] Camp SM, Ceco E, Evenoski CL, Danilov SM, Zhou T, Chiang ET (2015). Unique toll-like receptor 4 activation by NAMPT/PBEF induces NFkappaB signaling and inflammatory lung injury. Sci Rep.

[9] Sun BL, Sun X, Casanova N, Garcia AN, Oita R, Algotar AM (2020). Role of secreted extracellular nicotinamide phosphoribosyltransferase (eNAMPT) in prostate cancer progression: Novel biomarker and therapeutic target. EBioMedicine.

[10] Wang B, Hasan MK, Alvarado E, Yuan H, Wu H, Chen WY (2011). NAMPT overexpression in prostate cancer and its contribution to tumor cell survival and stress response. Oncogene.

